# Pulsierender Orbitatumor

**DOI:** 10.1007/s00347-020-01060-2

**Published:** 2020-02-19

**Authors:** C. S. Butsch, J. Heider, M. A. Brockmann, N. Pfeiffer, F. A. Ringel, E. Schwandt, K. A. Ponto

**Affiliations:** 1 Augenklinik und Poliklinik, Universitätsmedizin Mainz, Langenbeckstr. 1, 55131 Mainz, Deutschland; 2 Klinik für Mund‑, Kiefer- und Gesichtschirurgie, Universitätsmedizin Mainz, Langenbeckstr. 1, 55131 Mainz, Deutschland; 3 Klinik und Poliklinik für Neuroradiologie, Universitätsmedizin Mainz, Langenbeckstr. 1, 55131 Mainz, Deutschland; 4 Neurochirurgische Klinik und Poliklinik, Universitätsmedizin Mainz, Langenbeckstr. 1, 55131 Mainz, Deutschland

## Anamnese

**Figure 1:**
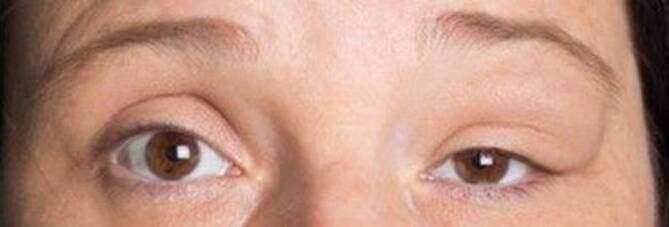

Eine 36-jährige Patientin stellte sich bei unklarer Raumforderung des linken Oberlides bzw. der temporal-superioren Orbita zur weiteren Abklärung in der interdisziplinären Orbitasprechstunde vor (Abb. 1). Anamnestisch bestanden seit etwa 6 Monaten eine progrediente Oberlidschwellung am linken Auge sowie eine subjektive Sehstörung linksseitig und ein Druckgefühl über dem Bulbus. Die ophthalmologische Anamnese sowie die Allgemeinanamnese waren bisher unauffällig.

## Klinischer Befund

Der Visus betrug ohne Korrektur beidseits 1,0, der intraokulare Druck 14 mm Hg am rechten Auge und 20 mm Hg am linken Auge. Am rechten Auge waren die vorderen Augenabschnitte reizfrei und regelrecht. Links lag eine temporal betonte Oberlidschwellung mit Ptosis, die etwa ein Drittel der Pupille bedeckte, vor (Abb. 1). Die Lidspaltenweite betrug am rechten Auge 10 mm und am linken Auge 6 mm in Primärposition. Palpatorisch zeigte sich ein praller Tumor im Oberlidbereich mit knotiger Konsistenz und tastbarer Pulsation. Die Pulsation ließ sich nicht auskultieren. Die Raumforderung war nicht verschieblich, und der Tastbefund ergab den Verdacht, dass die Läsion sich bis in die Tiefe ausdehnte. Ein relatives afferentes Pupillendefizit lag nicht vor. Die Hertel-Exophthalmometrie ergab einen seitengleichen Befund. Die hinteren Augenabschnitte waren beidseits unauffällig. Die orthoptische Untersuchung ergab eine regelrechte Augenstellung sowie eine freie Bulbusmotilität beidseits. In der statischen Perimetrie zeigte sich ein zur Ptosis passender superiorer Gesichtsfelddefekt am linken Auge.

## Bildgebung und Diagnose

**Figure 2:**
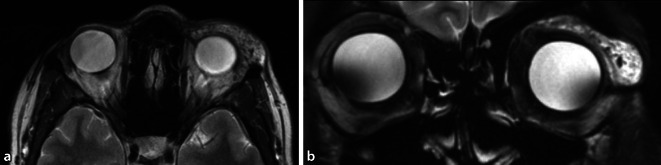

Es wurde eine Magnetresonanztomographie des Schädels und der Orbita nativ und nach Kontrastmittelgabe durchgeführt (Abb. 2). In den T2-gewichteten Sequenzen stellte sich nativ eine hyperintense, multilobulierte, glatt begrenzte Raumforderung ventral der Glandula lacrimalis dar. Nach Kontrastmittelapplikation zeigte sich ein kräftiges Enhancement der Raumforderung.

Es erfolgte eine Doppler- und farbkodierte Duplexsonographie zur Beurteilung der Durchblutungssituation („high-flow“ vs. „low-flow“). Diese ergaben eine deutliche Hyperperfusion mit schneller Strömungsgeschwindigkeit („high flow“) in den zuführenden Gefäßen.

## Wie lautet Ihre Diagnose?

Es ergab sich der dringende Verdacht auf eine arteriovenöse Malformation (AVM) gemäß der Klassifikation der International Society for the Study of Vascular Anaomalies (ISSVA) [3].

**Figure 3:**
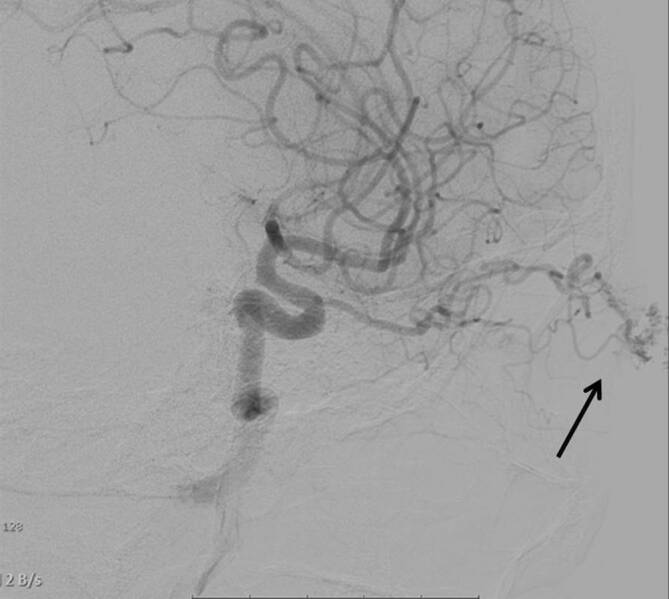

Zur Bestätigung dieser Verdachtsdiagnose und weiteren Evaluation erfolgte eine digitale Subtraktionsangiographie (DSA) der kraniozervikalen Gefäße. Es zeigte sich eine AVM mit größtenteils arterieller Versorgung über 2 Gefäße aus der A. ophthalmica sowie geringem, nur langsamem venösem Abfluss (Abb. 3).

## Doppler- und Indocyaningrün-Angiographie-gestützte Resektion

Nach Ausschluss endovaskulärer Therapieoptionen fiel die Entscheidung zur Doppler-gestützten chirurgischen Entfernung der AVM in toto mit intraoperativer Indocyaningrün(ICG)-Angiographie. Diese führten wir interdisziplinär mit Kollegen der Abteilung für Neurochirurgie durch. Differenzialtherapeutisch wurden die Sklerosierung der zuführenden Gefäße mit dem Ziel einer Schrumpfung der Raumforderung oder ausgedehntere chirurgische Verfahren diskutiert.

Eine erste ICG-Angiographie intraoperativ erfolgte nach Freilegung der vaskulären Malformation zur Identifizierung der zuführenden Arterien beim Anfluten des Farbstoffs sowie zur Darstellung der Flussverhältnisse im Nidus nach folgendem Schema: Es werden jeweils 2 ml der Indocyaningrün-Injektionslösung mit einer Wirkstoffkonzentration von 5 mg/ml intravenös injiziert, nachdem der ICG-Angiographie-Modus des Mikroskops aktiviert wurde. Auf einem schwarz-weißen Monitorbild sind dann das Anfluten, die Perfusion und das Auswaschen des weiß leuchtenden Farbstoffs in Echtzeit zu verfolgen, zusätzlich zeichnet das Mikroskop eine Videoschleife auf, die zur Beurteilung wieder und wieder abgespielt werden kann. Es können jedoch nur oberflächliche, zuvor im Mikroskop sichtbare Strukturen durch diese Methode beurteilt werden. Nach der Resektion kann eine ICG-Angiographie wiederholt werden, um die vollständige Ausschaltung des arteriovenösen Shunts zu dokumentieren.

Intraoperativ wurde zusätzlich eine Einweg-Mikrodopplersonde (® Mizuho Medical Systems Co., Tokyo, Japan, Surgical Doppler REF 0715002) verwendet, um den Blutfluss in den zu- und abführenden vaskulären Strukturen als Doppler-Signal hörbar zu machen. Hierfür wurde die angefeuchtete Spitze der Doppler-Sonde (Messtiefe 1 mm) direkt auf das zu beurteilende Gefäß aufgesetzt. Diese unkomplizierte, schnelle und jederzeit wiederholbare Untersuchung wird vor, während und nach Resektion der AVM wiederholt durchgeführt, wobei die Sonde möglichst im gleichen Winkel auf das jeweilige Gefäß aufgesetzt werden sollte.

## Arteriovenöse Malformation der Orbita

Für die Nomenklatur von Gefäßmalformationen des Kopf-Hals-Bereiches ist eine einheitliche Sprachregelung unerlässlich. Es hat sich daher eine Einteilung peripherer Gefäßerkrankungen nach der ISSVA-Klassifikation [3] etabliert, die auch für orbitale Prozesse als valide erachtet wird [5]. Im vorliegenden Fall bestand gemäß dieser Klassifikation eine solitäre AVM. In Abgrenzung zu vaskulären Tumoren und kombinierten vaskulären Malformationen werden solitäre AVM als simple, vaskuläre High-flow-Malformation kategorisiert. Die präziseste Diagnostik einer AVM erlaubt die DSA, in der das exakte Ausmaß des „Nidus“ (Ort der arteriovenösen Kurzschlüsse) mit allen zuführenden erweiterten arteriellen Ästen („feeder“) und Drainagevenen mit gleichzeitiger Erfassung der Hämodynamik dargestellt wird. AVM im Lid- und Orbitabereich können spontan oder in Folge eines Traumas auftreten [4]. Sie können mit kosmetischer Entstellung und funktionellen Einschränkungen wie Visusminderung und Motilitätseinschränkungen einhergehen.

Die chirurgische Exzision birgt das Risiko von Blutungskomplikationen bis hin zum Organverlust sowie einer weiteren Entstellung durch große Defekte. Nach Koagulation von Endarterien kann es postoperativ zu retinalen Gefäßverschlüssen kommen.

**Diagnose:** Arteriovenöse Malformation (AVM) gemäß der Klassifikation der International Society for the Study of Vascular Anaomalies (ISSVA)

Die chirurgische Resektion unter Zuhilfenahme der ICG-Angiographie wird heutzutage bei zerebralen AVM zur intraoperativen Identifikation der Gefäßarchitektur arterieller Feeder-Gefäße, drainierender Venen sowie AVM-Residuen eingesetzt [1, 7].

Die intraoperative Doppler-Sonographie ist ein weiteres Verfahren, die Gefäßarchitektur zu identifizieren und so eine vollständige Resektion unter maximaler Schonung des umliegenden Gewebes zu ermöglichen [6].

Der vorliegende Fall illustriert, dass durch die Kombination beider Verfahren das Komplikationsrisiko bei der Resektion dieser vaskulären Malformationen möglicherweise noch weiter reduziert werden kann. Durch unterschiedliche Stärken beider Methoden (bessere Darstellung der Gefäße innerhalb der Raumforderung mit der ICG-Angiographie und bessere Darstellung auch tiefer verlaufender Feeder-Gefäße) wird durch die Verwendung beider Techniken ein hohes Maß an intraoperativer Sicherheit erreicht. Den Autoren erschien der kombinierte Einsatz sowohl der ICG- als auch der Doppler-Navigation bei bestehender Anastomosierung der AVM mit Gefäßen aus der A. ophthalmica und damit drohendem Visus- und Organverlust durchaus gerechtfertigt.

Abhängig von den individuellen AVM-Merkmalen können unterschiedliche interdisziplinäre Herangehensweisen erforderlich sein. In der Literatur wurde über die Kombination der intravasalen Embolisation und der chirurgischen Exzision berichtet [2]. In unserem Fall konnte durch die interdisziplinäre Zusammenarbeit von Neuro- und Orbitachirurgen ein zufriedenstellendes Ergebnis erzielt werden. In jedem Fall sollte die Behandlung dieser Patienten in enger Zusammenarbeit hoch spezialisierter Fachrichtungen optimalerweise an einem interdisziplinären Orbitazentrum erfolgen.

## Differenzialdiagnosen

Basierend auf dem klinischen Befund allein, kamen neben einer Gefäßmalformation solide Tumoren in der Orbita und in der Tränendrüse infrage. Differenzialdiagnostisch kategorisiert die ISSVA-Klassifikation weiterhin Gefäßtumoren und Gefäßanomalien [3]. Hierbei wird zwischen vaskulären Tumoren und vaskulären Malformationen unterschieden. Bei Gefäßtumoren besteht eine tatsächliche Endothelzellproliferation. Davon abzugrenzen sind Gefäßmalformationen, die einen normalen Endothelzellzyklus aufweisen. Die vaskulären Malformationen werden weiter in einfache und kombinierte (z. B. arteriös, venös, lymphatisch, kapillär) unterschieden. Ebenso treten Assoziationen von Gefäßmalformationen mit anderen Anomalien (z. B. Hyperplasien des Weichteilgewebes) in Form von Syndromen auf („malformations associated with other anomalies“).

## Prozedere

**Figure 4:**
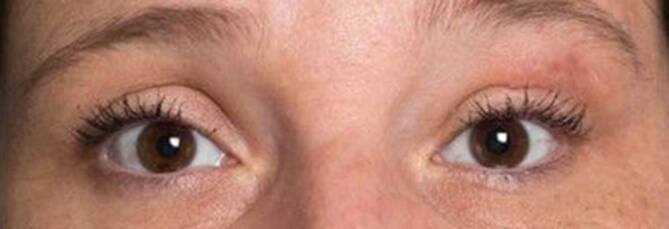

**Figure 5:**
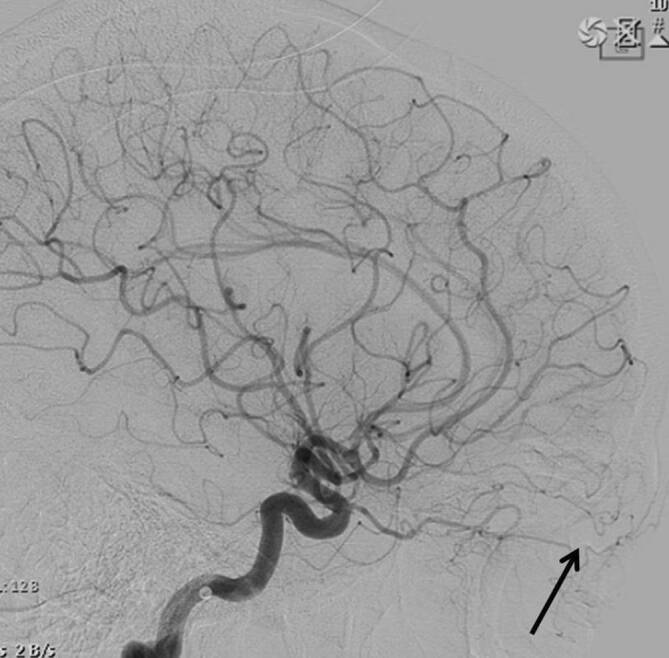

Eine Verlaufs-DSA (Abb. 4) 1 Monat postoperativ bei der beschwerdefreien Patientin (Abb. 5) zeigte eine vollständige Entfernung der AVM.

## Schlussfolgerung

Die AVM ist eine seltene, aufgrund der vielfältigen potenziellen Komplikationen gravierende orbitale Raumforderung. Die Diagnose und Spezifikation erfolgen mittels hoch spezialisierter radiologischer Verfahren. Die Doppler- und ICG-navigierte Resektion stellen eine sichere und gewebeschonende Therapieoption dieser Gefäßveränderungen dar. Die ICG-navigierte Chirurgie bleibt interdisziplinären Settings an größeren Zentren vorbehalten. Größere interdisziplinäre Orbitazentren verfügen zwar häufig über neurochirurgische Mikroskope, aber auch diese sind in aller Regel nicht mit ICG-Modus ausgestattet, da die Anschaffungskosten hierfür (bei relativ wenigen Indikationen auch an spezialisierten Zentren) verhältnismäßig zu hoch sind. Mikroskope neurochirurgischer Abteilungen verfügen in aller Regel über die erforderliche Ausstattung.

Das verwendete Gerät für die intraoperative Doppler-Untersuchung mit Einweg-Mikrodopplersonde (® Mizuho Medical Systems Co., Surgical Doppler REF 0715002) hat sehr geringe Anschaffungs- sowie laufende Kosten für Verbrauchsmaterial und ist sehr einfach in der Anwendung. Außerdem kommt es für verschiedene andere Indikationen in der Orbita- und okuloplastischen Chirurgie infrage (z. B. für Biopsie der A. temporalis, Tumorchirurgie, rekonstruktive Chirurgie). Die intraoperative Doppler-Untersuchung stellt damit eine Bereicherung der chirurgischen Möglichkeiten in der Augenmedizin dar, auch außerhalb von interdisziplinären Settings.

## Fazit für die Praxis

- Für die Nomenklatur von Gefäßmalformationen des Kopf-Hals-Bereiches hat sich eine Einteilung peripherer Gefäßerkrankungen nach der ISSVA(International Society for the Study of Vascular Anaomalies)-Klassifikation etabliert, die auch für orbitale Prozesse als valide erachtet wird.
- Die Diagnose und Spezifikation der arteriovenösen Malformation (AVM) erfolgen mittels hoch spezialisierter radiologischer Verfahren.
- Die Doppler- und ICG-navigierte Resektion bieten eine sichere und gewebeschonende Therapieoption.
